# Pollution of Flooded Arable Soils with Heavy Metals and Polycyclic Aromatic Hydrocarbons (PAHs)

**DOI:** 10.1007/s11270-014-2145-0

**Published:** 2014-09-17

**Authors:** Tomasz Ciesielczuk, Grzegorz Kusza, Joanna Poluszyńska, Katarzyna Kochanowska

**Affiliations:** 1 Department of Land Protection, University of Opole, 22 Oleska St., 45-052, Opole, Poland; 2 Institute of Ceramics and Building Materials, Oswiecimska 21 St., 45-641, Opole, Poland

**Keywords:** Soil, Flood, Heavy metals, PAHs

## Abstract

Soils that are exposed to floodwaters because of shallow groundwater and periodical wetlands are, to a large extent, exposed to contamination by organic and inorganic compounds. These are mainly compounds that have drifted along with the inflow of heavily laden floodwater and are produced within the soil profile by the anaerobic transformation of organic matter. Heavy metals and polycyclic aromatic hydrocarbon (PAH) compounds are absorbed by the soil of the floodwaters, and moving in the soil profile, they pose a threat to groundwater. What is more, after a flood, they may be absorbed by the crops. This paper focuses on the effects of Odra River (Poland) floods, heavy metals, and PAHs on soil and the possibilities of the migration of these pollutants into the soil profile. In the tested sludge samples of floodwater and soil, there were no abnormal concentrations of heavy metals, but the flooding time positively affected the amount listed in the test samples. Concentrations of PAHs increased, but they also exceeded the standards for arable soils in the case of single compounds.

## Introduction

Severe and prolonged rainfall leads to water levels rising in rivers not only in mountainous regions but also in the lowlands. River surge, resulting in permeating or flooding, causes long-term negative effects to the environment. Sustained flooded soil, even for a short time, leads to the displacement of air from the soil pores and the creation of anaerobic conditions in the profile. The saturation stage during flooding includes all levels of genetic soil, resulting in marginal amounts of oxygen being absorbed in the soil profile. This entails the death of plants that are usually not suited for prolonged submergence underwater; it also destroys soil fauna as well as influences the chemical composition of the soil. The organic compounds in the soil of the incoming water are absorbed by the sorption complex, contributing in particular to the pollution of the humus level (Maliszewska-Kodyrbach et al. 2008). The composition of the soil has a huge impact on the amount of the substance being absorbed. If it has been previously contaminated with organic compounds of anthropogenic origin, such as tar or soot, the sorption of pollutants may increase (Motelay-Massei 2004). In addition, the dying roots of submerged plants are subjected to microbial decomposition with the evolution of hydrogen sulfide, methane, and other compounds including polycyclic hydrocarbons. Anaerobic conditions can significantly cause the decomposition of biomass—up to a sixfold increase in the amount of compounds from the group of polycyclic aromatic hydrocarbons (PAHs) in relation to the amount present in the soil before flooding (Oleszczuk et al. 2007). Absorbed with floodwater or produced in the process of microbial degradation PAHs compounds because of the occurrence in the floodwaters, hydrophilic compounds act as solvents and migrate through the soil profile along with the moving water. This is especially dangerous in the case of direct contact with the groundwater aquifers, which are used as sources of drinking water. The bioavailable fraction of PAHs can be absorbed by plants that will grow in the area covered by floodwater recession.

Floodwater includes not only significant loads of suspensions and nutrients but also large amounts of organic compounds, often harmful or toxic (Czerniawska-Kusza et al. 2006; Witt and Siegel 2000). This contamination by the floodwaters is washed away from roads, exercise yards, warehouses and petrol stations, places where solvents and pesticides are disposed, and landfills and domestic septic tanks that collect household sewage (Poluszyńska 2012). Pollution carried by floodwater can be divided into two major groups: the first group includes primarily inorganic substances that contain heavy metals and nutrients, which are retained by physical or chemical sorption during the process of migration into the soil profile and finally enrich the flooded soil (Maliszewska-Kodyrbach et al. 2012). The other group consists of the organic compounds present in the form of the predecomposed remains of plant and animal tissues derived from municipal waste and organic matter contained in compost, manure, slurry tanks, and septic tanks as well as organic compounds that may have toxic properties (e.g., omitted refining lubricants or substances with irritant properties, such as, alcohols and hydrocarbons, including aliphatic and monoaromatic hydrocarbons) (Kluska 2004; Laskowski et al. 2005). In addition, these waters contain compounds belonging to the so-called persistent organic pollutants (POPs), such as PAHs, polychlorinated dibenzodioxins (PCDDs), polychlorinated dibenzofurans (PCDFs), polychlorinated biphenyls (PCBs), and polychlorinated terphenyls (PCTs) (Witt and Siegel 2000). In between flooding, the soil is exposed, although to a much lesser extent, to the contamination of POPs (including PAHs), resulting in the precipitation of dry deposition. Many sources of these compounds, in combination with the significant dispersion, result in PAHs that are listed in all areas of the environment (Carlstrom and Tuovinen 2003; Chen et al. 2004; Wilcke et al. 2004). This may be natural (e.g., volcanoes, forest fires, and the distribution of biomass) or anthropogenic (e.g., fuel combustion) (Thiele and Brummer 2002). This group includes more than 200 compounds; however, 16 of them with particularly toxic (including carcinogenic) properties were chosen for monitoring. Not only the floodwaters but also the crops’ organic fertilization and sedimentation to the soil surface dust can add significant amounts of metals and POPs (Wilcke et al. 2004; Laskowski et al. 2005). In addition, organic fertilization with manure or compost, especially sewage sludge, also acts as a soil enrichment process, among others, in polycyclic hydrocarbons (Brandli et al. 2005; Oleszczuk 2006; Weber et al. 2007). Content of listed PAH compounds in soils accelerate anaerobic conditions (as a result of organic matter fermentation in the soil), often occurring with high groundwater levels, especially during a flood, when the waters cover the soil for a long time (Włodarczyk-Makuła and Janosz-Rajczyk 2004). Entering the soil, allochthonous metal ions and PAHs are adsorbed mainly in the humus layer (Kluska 2004). Organic matter present in amounts greater than 0.1 % is largely responsible for the sorption of pollutants (Yang et al. 2008). However, the compounds of PAHs, despite the relatively low solubility in water, accompanied by substances (e.g., humic acids) may migrate along the direction of water flow into the profile and then be taken up by the plants or enter aquifers, causing contamination, especially in the case of light soils (Ciesielczuk et al. 2006a, b; Wilcke et al. 1999). The article focuses on the presence of heavy metals and PAHs in soils subjected to short and long processes of complete inundation by floodwater as a factor that could eliminate the soil from cultivation crops intended for direct consumption.

## Material and Methods

### Research Area

**Fig. 1:**
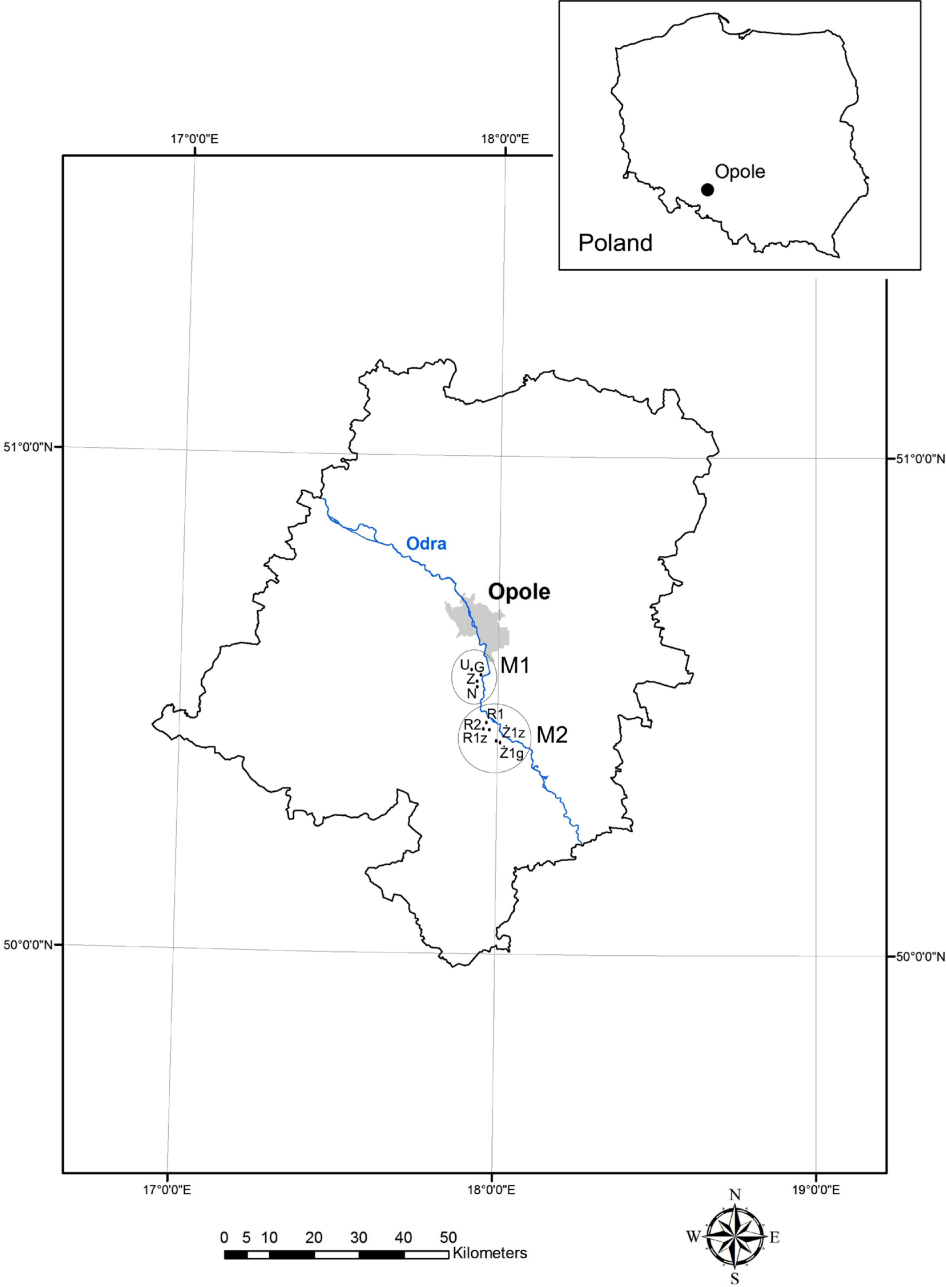
Sampling area localization

An area located in the southern part of the city of Opole where floodwaters have passed (M1) was chosen for the research. Furthermore, an area near Krapkowice (M2) was analyzed (Fig. 1). Both areas are located in the Opole Voivodeship (southern Poland). The two areas studied were covered by the flood from Odra River. In 1997 occurred the most dangerous flood on Odra River in 20th century (Dubicki et al. 2005). In case of flood phenomenon, the main sources of heavy metals and PAHs are agricultural soil leaching, sewage treatment plants, landfill leachates, and industrial areas. Also, PAHs can be generated in the anaerobic decomposition of biomass contained in flood sediments. The sampling at the area M1 was carried out on May 28, 2010, from topsoil to a depth of 0.2 m. The samples were taken from four locations: N (mineral silt), G (organic-mineral silt from the area briefly flooded), Z (long-cultivated soil, flooded for a long time), and U (0–0.2 m level of arable land outside the flooded area [background]). The period of retention of floodwaters in the study area was about 1 day for sample N, about 2 to 4 days for sample G and 2 weeks in the case of sample Z. Sample U (control) was taken from arable soil about 60 m from the maximum flood range (Table 1).

**Table 1:** Sampling site characteristics of area M1

	N	E	Altitude (m)
N	50°37′29.21″	17°57′67.10″	154
G	50°37′28.49″	17°57′45.81″	153
Z	50°37′26.44″	17°56′51.59″	151
U	50°37′31.25″	17°57′10.82″	156

The sampling in the area M2 (Table 2) was performed on May 29, 2010, from arable soil to a depth of 0.2 m. The samples were taken in Rogów Opolski (R1 and R2 [silt mineral] and R1z [humus level of arable soil (background)]) and Żużela (Ż2g [mineral silt] and Ż1z [humus level of arable soil (background)]). An average sample was obtained by homogenizing 12 subsamples taken within each sampling area, which covers about 100 m^2^.

**Table 2:** Sampling site characteristics of area M2

	N	E	Altitude (m)
R1	50°30′48.88″	17°56′53.81″	158
R1z	50°30′48.75″	17°56′44.67″	157
R2	50°30′52.03″	17°57′30.38″	160
Ż1z	50°26′27.67″	18° 00′39.24″	162
Ż2g	50°26′28.68″	18° 00′43.03″	161

### Methods

In soil samples, the following parameters were determined: pH, electrolytic conductivity (EC), and the content of organic matter (methodology according to Polish standards). The contents of calcium, sodium, potassium, and lithium were determined in wet mineralizates through the flame emission spectrometry (FES) method using the BWB-XP apparatus. The contents of the other analyzed metals (zinc, copper, chromium, nickel, lead, cadmium, and manganese) were determined using the Thermo Scientific iCE 3500 Atomic Absorption Spectrometer after the microwave-assisted wet mineralization in aqua regia using the MARS-X device. Mercury content was determined in solid samples through atomic absorption spectrometry with the AMA-254 apparatus. Analytical procedure quality was checked with “LRM soil 1”—certified reference material (Gdańsk University of Technology, Poland).

A PAH analysis was carried out in a few steps. First of all, fresh samples were dried at room temperature using anhydrous sodium sulfate (POCH) (Ciesielczuk et al. 2006a, b). Extraction was carried out through a DCM/hexane (gas chromatography grade) mixture in a ratio of 1:9 (*v*/*v*) in the extraction device fexIKA (Laskowski et al. 2005; Kluska 2004). Before GC analysis, the extracts were purified by aluminum oxide (Aldrich) on glass tubes (Wilcke et al. 1999). Inspissated eluates were analyzed through the gas chromatography–mass spectrometry (GC-MS) method (Shimadzu GC 17A with MS-QP5000) on the capillary column VF-5ms (30 m, 0.25 mm i.d., and 0.25 μm). The temperature of the chromatograph injector was 300 °C while that of the detector was 320 °C. The oven temperature program involved the following: 80 °C at 8 min, heating 10 °C/min to 270 °C and heating 2 °C/min to 300 °C. The detector current was at 1.2–1.4 kV. In each sample, 16 single compounds, which are recommended for monitoring by U.S. Environmental Protection Agency (U.S. EPA), were determined: naphthalene (Naf), acenaphthylene (Acy), acenaphthene (Ace), fluorene (Flu), phenanthrene (Phe), anthracene (Ant), fluoranthene (Flt), pyrene (Pyr), benzo[a]anthracene (B[a]ant), chrysene (Chr), benzo[b,k]fluoranthene (B[b,k]flt), benzo[a]pyrene (B[a]p), dibenzo[a,h]anthracene (D[a,h]ant), indeno[1,2,3-c,d]pyrene (Ind[123]p), and benzo[g,h,i]perylene (B[ghi]per). The flow of a carrier gas (He) was adjusted to 1 cm^3^/min. Certified PAH standards (US-106 N 2,000 μg/cm^3^ of each compound, Ultra Scientific, USA) were used in order to determine the calibration curve. Recovery levels for this procedure were low for naphthalene (57–66 %) and higher (73–92 %) for the rest of the individual PAHs. The recovery procedure was based on dry samples of PAH reference materials: “ERM-CC013a” (BAM, Berlin, Germany) and “LRM soil 1” (University of Technology, Gdańsk, Poland). The detection limits ranged between 0.05 and 0.1 μg/kg (dry weight) for particular PAHs. The uncertainty of the results was calculated as a standard deviation value. In order to single out the petrogenic compounds, the PAH contents of ANT/(ANT + PHE), BaA/(BaA + CHR), and FLA/(FLA + PYR) in liquid and solid fuel combustion were calculated.

## Results and Discussion

### Granulometric Characteristics of Investigated Samples

A significant quantity of the skeleton parts indicates silt a short distance from the hole it was taken from. However, a large part of fine fractions, in accordance with the law of gravity, indicates a relatively long transport of mineral matter before sedimentation. Sample N is well-sieved sand devoid of large fractions—skeleton elements and even the smallest silt and clay, which is characteristic of mineral sediments being transported by water in a relatively short distance from the place where they were taken from (Table 3). Sample G and sample N are mineral materials deposited on farmland during the floods in 2010. It is shown by the absence of colloidal clay fractions and a very small fraction of the dust. A relatively large fraction of backbone >2 mm (11 %) indicates a short transportation through a mineral silt fence.

**Table 3:** Granulometric composition of samples from the areas M1 and M2 (%)

		Share of fractions (mm)					
		>2 mm	<2 mm	Σ sand 2–0.05 mm	Σ fractions 0.05–0.002 mm	Σ fractions <0.002 mm	Group and subgroup
M1	N	0	100	99	1	0	cs
	G	11	89	98	1	1	cs
	Z	2	98	76	20	4	ls
	U	5	95	86	10	4	ls
M2	R1	0	100	95	4	1	cs
	R1z	6	94	85	12	3	ls
	R2	0	100	93	5	2	cs
	Ż2g	0	100	98	1	1	cs
	Ż1z	8	92	69	22	9	sl

*cs* coarse sand, *ls* loamy sand, *sl* sandy loam

Sample Z has a granulometric composition characteristic of an arable humus alluvial river. The soil was formed by the alluvial deposition of mineral masses, and then through proper agriculture, this level has been enriched with humus (Table 4). Sample U, similar to sample Z, is a natural soil (alluvial soil). In all tested samples, the reaction was close to neutral (6.05–7.40). EC was generally low, except in sample Z, which was flooded for a long time. All the tested samples were the deposits of low organic matter content.

**Table 4:** Main parameters of samples from the areas M1 and M2

	M1				M2				
	N	G	Z	U	R1	R1z	R2	Ż2g	Ż1z
Reaction (pH)	6.45	7.07	7.30	7.05	6.30	6.05	6.46	7.40	6.21
Electrolytic conductivity (μS/cm)	20.8	70.7	172.6	67.5	21.2	20.2	25.1	79.5	18.0
Organic matter (%)	0.31	0.91	4.51	3.29	1.56	1.45	2.80	2.02	0.37
Organic carbon (%)	0.13	0.48	3.63	1.58	0.22	0.35	1.24	1.34	0.06
CaO (mg/kg [dry matter])	235.0	253.2	937.3	551.2	126.2	45.5	209.8	301.4	< 1.5
Na_2_O (mg/kg [dry matter])	< 3.0	13.1	142.3	68.7	203.0	218.5	202.8	313.5	140.2
P_2_O_5_ (%)	< 0.05	0.15	0.87	0.40	< 0.05	0.11	0.18	0.09	< 0.05
K_2_O (mg/kg [dry matter])	258.6	244.4	2685	1176	1992	1190	2723	2851	612.7

During flood phenomenon, concentrations of nutrients rise in floodwater. In particular, high concentrations of P-PO4 were noted (Fenske et al. 2001). In case of long contact with soil, phosphates can be absorbed in the top (organic) layer of soil, which finally leads to higher values of phosphorus noted in samples Z and R2. Also, the contents of potassium and calcium compounds correspond well with organic matter content. Globally, the highest values were noted in sample Z flooded for a long time. The sampling site where sample Z was taken had no outflow because of riverbanks. So most likely, a lot of water transpired, and concentrations of all components rose. Remaining water migrated through a soil profile, and a lot of compounds were stopped in a mechanical or chemical manner. A similar situation was noted in the case of sample Ż2g. The highest values of pH were also noted in both samples as a result of concentrations of alkaline metals.

### Law Regulations

The Ministry of Environment established a regulation on September 9, 2002, on the standards for soil quality, which require the determination of metals such as zinc, copper, lead, nickel, chromium, cadmium, and manganese. Tested soil and deposits contain small amounts of heavy metals characteristic of uncontaminated soil (Karczewska et al. 2009; Hernandez et al. 2003; Rosik-Dulewska Cz et al. 2008; Steinnes et al. 2005). The regulation also requires the determination of only several PAHs (Table 5) out of the 16 recommended by the U.S. EPA to monitor. The most stringent standards apply to the soil group A and the 0–0.3-m soil layer of group B. For these soils, the concentration level is 0.1 mg/kg (dry matter) for each PAH and 1 mg/kg (dry matter) for the sum of nine compounds. The exception is benzo(a)pyrene, in which the maximum level was set at 0.02 mg/kg (dry matter) and 0.03 mg/kg (dry matter), respectively, of soil from group A and group B (Soil Quality Polish Regulation from September 9 2002). However, many researchers expand the list of test compounds, usually focusing on the last six from U.S. EPA list, which are characterized by the strongest toxicity (Chen et al. 2004; Wilcke et al. 2004; Laskowski et al. 2005; Kluska 2004).

**Table 5:**
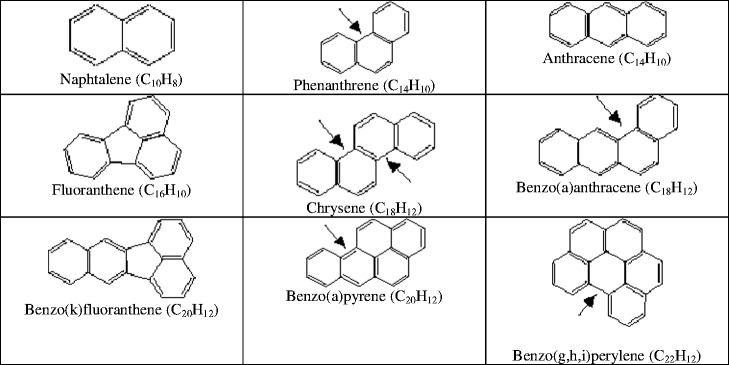
Compounds from the PAH group for monitoring soil samples (Soil Quality Polish Regulation)

### Chemical Composition

Floodwater could be an important source of some heavy metals. In particular, lead, cadmium, and mercury concentrations in Odra River rose during the flood, but only 40–60 % of metals found in flooded soils were available for plants (Fenske et al. 2001; Helios et al. 2005). The recorded amounts of metals did not exceed the permissible limits; however, they were highly changeable (Table 6). The lowest concentration was recorded in the case of material originating from a flood embankment caving away (sample N). This can be explained by the small sorption complex of the sample. Slightly higher (1.48–3.10 times in comparison to sample N) amounts were recorded in a sample from native soil from a short period of flooding (sample G). The highest amounts of metals (12.4–171 times compared with a sample N) were recorded in long-term flooded soil (sample Z), covered with organic-mineral deposit, which confirms the mechanism described above. Agricultural soil contained 4.3–17.9 times more metal than sample N, but even the most contaminated of the respondents (sample Z) do not exceed the permissible limits.

**Table 6:** Contents of heavy metals in investigated samples from the areas M1 and M1 (mg/kg [dry matter])

	M1					M2				
	N	G	Z	U	R1	R1z	R2	Ż2g	Ż1z	Limit (31)
Zn	3.74	11.58	156.0	66.99	14.22	18.45	111.8	47.28	7.40	300
Cu	0.44	1.16	75.29	4.80	5.35	3.47	1.68	5.56	2.27	150
Ni	0.90	1.58	11.20	3.90	12.67	9.15	14.85	16.13	3.20	100
Cd	< 0.02	< 0.02	0.39	< 0.02	< 0.02	0.02	0.20	0.04	0.02	4
Pb	1.74	3.37	22.01	15.19	4.74	6.44	12.59	6.07	0.96	100
Cr	0.75	1.83	13.72	6.01	4.51	3.19	6.40	6.95	0.66	150
Mn	32.88	48.64	409.6	241.0	77.60	191.0	347.4	297.3	39.88	–
Hg	0.0070	0.0260	0.0759	0.0481	0.0351	0.0394	0.0350	0.0709	0.0189	2

In the samples from the case of M2, recorded amounts of heavy metals were high and similar to those observed in the case of M1. None of the samples analyzed contained abnormal amounts of these elements. The highest value was recorded in sample Ż2g (379.4 mg/kg [dry matter]) and sample R2 (495.0 mg/kg [dry matter]), while the lowest amounts of metals were recorded in sample Ż1z (only 54.4 mg/kg [dry matter]) These observations correspond well with other data about low risk from metals after phenomenal floods on arable soil areas (Maliszewska-Kodyrbach et al. 2012; Vácha et al. 2003). Other data were obtained on this area by other authors (Helios et al. 2005), where high concentrations of lead, cadmium, copper, and zinc in soil cores were found, but the highest values were found in deep layers (over 100 cm) of ground. Globally, the distributions of metals in all analyzed samples were similar (correlation coefficient of 0.97–0.99). Floodwater contained a lot of suspended organic and mineral matter (high turbidity), which led to the remobilizing of heavy metals (Fenske et al. 2001). However, the obtained results of metal concentrations were not as high as expected, but the long stagnation of floodwater is a risk from the rise of heavy metal content in soil because of water evaporation and metal sorption processes.

**Fig. 2:**
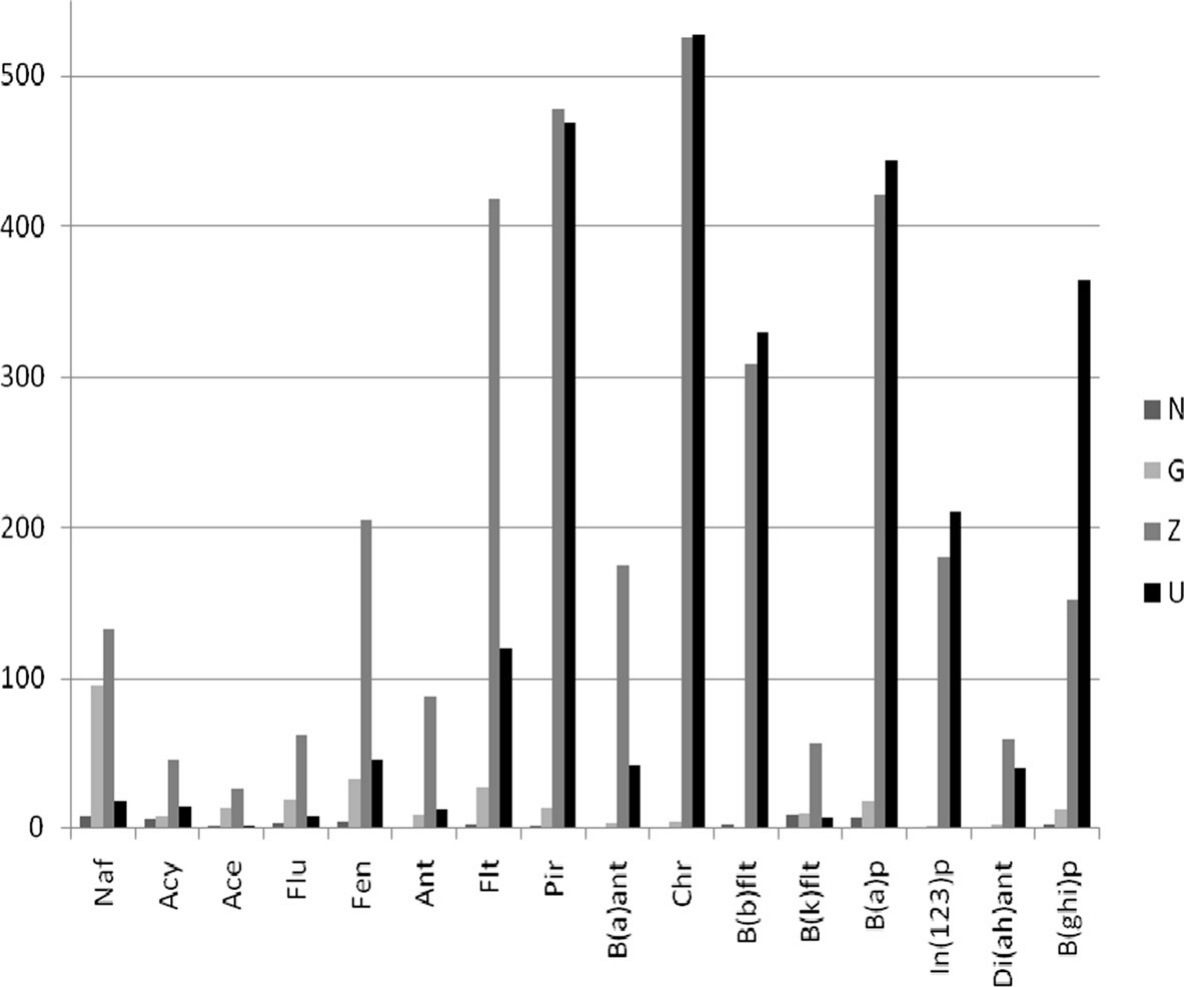
Content of 16 PAH compounds in investigated soils of the area M1 (μg/kg [dry matter])

Compounds of the PAH group never occur individually in the environment, but always in the form of compound mixtures. Their presence is widespread in every component of the environment, but given their properties, their excessive amounts can be harmful to living organisms (Oleszczuk 2006). The naturally and anthropogenically changing content of the soil is comprised within very wide limits (Table 7) and depends on the composition of the soil, the climate, the pollution level, and the method of its exploitation. Soils with a developed humus level contain increased amounts of these compounds. In particular, flooded deposits have significantly higher amounts of accumulated PAH, even reaching 5–8 mg/kg (dry matter) for individual compounds, particularly in the case of phenanthrene and fluoranthene (Witt and Siegel 2000). This also applies to hydromorphic soils with high groundwater and wetlands, where recorded amounts are in the range of 0.18–0.83 mg/kg (dry matter) for floodplain and 0.63–0.96 mg/kg (dry matter) for mangrove sediments (Thiele and Brummer 2002; Zheng et al. 2002; Atanassova and Brummer 2004). In particular, however, it applies to flooded soils. Two parallel phenomena take place then: (1) the sorption of compounds from the inflow of water and (2) their production in the process of soil biomass fermentation. The sorption of organic impurities is strongly dependent on the composition of the soil. The soils of the studied areas are located in the areas where the background prevalence of PAHs in agricultural soils are high and may exceed 1 mg/kg (dry matter) for the total amount of nine compounds (Maliszewska-Kodyrbach et al. 2008). The PAH quantities recorded in the soils of the area M1 are high, in particular the content of fluoranthene, of which permissible content for the soil type B (Fig. 2) exceeded more than fourfold, chrysene over fivefold, and benzo(a)pyrene more than tenfold. In the case of B(b)flt, B(a)p, In(123)p, and B(ghi)p, the largest quantities of these compounds were observed in cultivated soils free from floodwater coverage. In this case, it may be related to the regular organic fertilization with manure. Anaerobic decomposition of organic matter could lead to the increase in PAH concentration, but only in the case of samples Z (M1) and R1z, R2, and Ż2g (M2) because of organic matter content or the sorption processes during long contact with floodwater. Elevated levels of PAH in the samples may cause an increase in the ecotoxicity of the flooded area, leading to the withdrawal of more sensitive species (Czerniawska-Kusza and Kusza 2011). The highest total amount of the PAHs of nine compounds was observed as expected in long-flooded soils (sample Z), more than 2.172 mg/kg (dry matter), and in arable soil (sample U), 1.576 mg/kg (dry matter), which is higher than the amount usually observed in the terraces and flood mangrove areas and therefore areas with disturbed air-water relations in the soil (Elhottova et al. 2006; Thiele and Brummer 2002; Zheng et al. 2002; Atanassova and Brummer 2004). This shows that both flood and organic fertilizers are comparable sources of the soils’ PAHs. The observed values in samples U and Z are six to ten times higher than the national level of Σ9 PAHs 0.260 mg/kg (dry matter) (Maliszewska-Kodyrbach et al. 2012).

**Table 7:** Sum of the 16 PAH compounds in soils

	Sampling site characteristics	Sum of 16 PAH (μg/kg [dry matter])	Soil
1.	Industrial area (Motelay-Massei et al. 2004)	5,650	Urban soil
2.	Bangkok City, Thailand (mean value *n* = 30) (Wilcke et al. 1999)	129.2	Hydromorphic (0–5 cm)
3.	University of Bonn experimental field (Atanassova and Brummer 2004)	200	Hydromorphic (0–5 cm)
4.	University of Bonn experimental field (Thiele and Brummer 2002)	249.6	Hydromorphic (0–5 cm)
5.	Agriculture field, 10 m from road (Laskowski et al. 2005)	1,241–15,485	Arable soil (0–20 cm)
6.	Hangzhou City, China (Chen et al. 2004)	59.7–615.8	Urban soil (0–5 cm)
7.	Nonpolluted arable soil (Mazur et al. 2006)	63.3	Arable soil (0–25 cm)
8.	Nonpolluted arable soil (Oleszczuk et al. 2007)	17.5	Sandy loam
9.	Brazilian savanna (Wilcke et al. 2004)	7,030	Mineral soil
10.	Floodplain terrace of Moselle River (Yang et al. 2008)	24,700	Alluvial soil

**Fig. 3:**
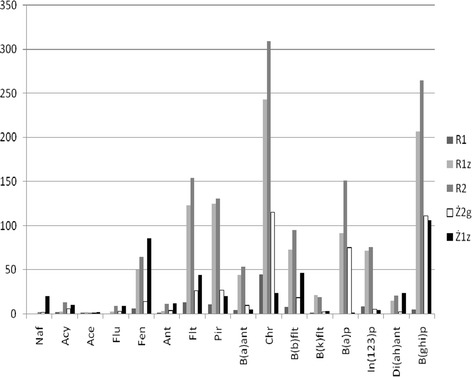
Content of 16 PAH compounds in investigated soils of the area M2 (μg/kg [dry matter])

In the case of the M2 object, the compounds of PAHs were occurring in smaller amounts compared with the M1 object (Fig. 3). Exceeding the permissible concentrations of the samples was recorded mainly for R1z, R2, and, exceptionally, (three compounds) Ż2g. The highest values, similar to the M1 object, were found for chrysene (Chr) (2.4–3.1 times exceeding the permissible values stated in the regulation) and B(ghi)p, where the exceeding values were 2.1–2.6 times. Lower concentrations were observed for B(a)p; however, because of a more stringent standard in this case, exceeding values above the allowable concentrations were 2.5–5.0 times. Nevertheless, it should be noted that the recorded amounts of PAHs were little, and only in one case (sample R2) did the total amount of these compounds slightly exceed the norms. The recorded amounts of these compounds in the samples of M2 object are commonly found in both urban areas and arable land (Wilcke et al. 2004). Low amounts of PAHs are probably caused by the nature of the mineral deposits, which results in a small sorption surface and a minimal organic matter content of less than 3 %. The calculated ratios of individual compounds indicate the anthropogenic character (derived mainly from the combustion of liquid and solid fuels) of determined PAHs. In this case, the decomposition of organics in low oxygen content did not hold much significance in the effect of low organic matter content. However, in samples Z and R2, this phenomenon could lead to PAH concentrations.

The creation of PAHs in the process of biomass decomposition can occur in all soils, but prolonged flooding intensifies this process. This is demonstrated by a model experiment, where after-sewage and distilled water were used in the process of flooding (Oleszczuk et al. 2007). When the water was poured, the changes in the prevalence of PAHs in the soil were investigated. In both cases, the observed increases in the PAH content of the experiment up to the 28th day were 28 and 38 % for distilled water and after-sewage water, respectively. After this time, there was a decrease in the content of the tested compounds. However, the behavior of individual groups of compounds varied. The strongest concentration growth was recorded for 5 and 6-ring PAHs. In the case of the “flood” made with distilled water, the final content of PAH (56 days) was less than 11–35 % compared with the beginning of the experiment. However, in the case of the “flood” made with after-sewage water, the final concentrations of the test compounds were higher than the initial, from 11 to 130 %. This points out the negative impact of flooding any soil with contaminated water (e.g., flood) by introducing substances that can increase the solubility and therefore the bioavailability of PAHs. Similar concentration dynamics (initial increase and then decrease) were recorded during the anaerobic incubation of sewage sludge (Włodarczyk-Makuła et al. 2003).

Model studies were also carried out by incubating the argillaceous soil under anaerobic conditions during the period of 730 days (Thiele and Brummer 2002). The increase in concentration was varied and ranged from 139 % for benzo(b)fluoranthene to 238 % for benzo(a)anthracene. In the case of lighter compounds, the increase in concentration was smaller, and even in the case of phenanthrene, there was a decline in its concentration levels. Pure plant material was incubated in a similar way, reaching a maximum rate of increase in concentration, which recorded 674 % for chrysene. This experiment shows that the decomposition of plant residues with oxygen deficiency leads to increased levels of PAHs by several times. A similar phenomenon was observed in glial soils with high groundwater level (Atanassova and Brummer 2004). Extremely reductive conditions at levels less than 175 cm result in generating benzo(g,h,i)perylene and dibenz(a,h)anthracene, as long as there are humic acids and biodegradable organic matter. PAHs also arise with smaller amounts of rings, such as naphthalene, acenaphthene, and phenanthrene. A huge impact on the amount of absorbed organic pollutants of floodwater has an original composition and pollution of the soil. If the soil is contaminated by the products of anthropogenic origin (e.g., tar, soot, ash), the amount of absorbed impurities and, among them, the compounds belonging to PAH will be higher (Oleszczuk et al. 2007; Yang et al. 2008). In the case the organic matter content in analyzed soil samples was low, microbial anoxic decomposition resulted in a higher PAH concentration, which was not observed and what was mentioned above.

## Conclusion

The flooding deposits and arable soils from the M1 (Opole) and M2 (Krapkowice) objects that were tested were characterized by relatively low heavy metal content, not exceeding permissible limits in any of the analyzed samples in this study. The reason for this was the low organic matter content and a significantly high content of coarse fractions resulting in a limited absorptive surface. Therefore, it seems that there is no ecotoxic impact from the analyzed elements. However, in several cases (samples Z, U, R1Z, R2, and Ż2g), pollution caused by the compounds from the PAH group was recorded. High amounts were reported particularly for chrysene, benzo(a)pyrene, and benzo(g,h,i)perylene, which can result in large amounts of these compounds to be detected in plants that will be grown in the area covered by the flood. A high content of PAH compounds in nonflooded arable soil (sample U) shows that both flood and organic fertilizers are comparable sources of soils’ PAHs.
